# Exoenzymes as a Signature of Microbial Response to Marine Environmental Conditions

**DOI:** 10.1128/mSystems.00290-20

**Published:** 2020-04-14

**Authors:** Manoj Kamalanathan, Shawn M. Doyle, Chen Xu, Amanda M. Achberger, Terry L. Wade, Kathy Schwehr, Peter H. Santschi, Jason B. Sylvan, Antonietta Quigg

**Affiliations:** a Department of Marine Biology, Texas A&M University at Galveston, Galveston, Texas, USA; b Department of Oceanography, Texas A&M University, College Station, Texas, USA; c Department of Marine Science, Texas A&M University at Galveston, Galveston, Texas, USA; d Geochemical and Environmental Research Group, Texas A&M University, College Station, Texas, USA; University of Hawaii at Manoa

**Keywords:** exoenzymes, nutrients, oil, microbial communities, polysaccharides, nutrient transport

## Abstract

Microbial heterotopic metabolism in the ocean is fueled by a supply of essential nutrients acquired via exoenzymes catalyzing depolymerization of high-molecular-weight compounds. Although the rates of activity for a variety of exoenzymes across various marine environments are well established, the factors regulating the production of these exoenzymes, and to some extent their correlation with microbial community composition, are less known. This study focuses on addressing these challenges using a mesocosm experiment that compared a natural seawater microbial community (control) and exposed (to oil) treatment. Exoenzyme activities for β-glucosidase, leucine aminopeptidase (LAP), and lipase were significantly correlated with dissolved nutrient concentrations. We measured correlations between carbon- and nitrogen-acquiring enzymes (*β*-glucosidase/lipase versus LAP) and found that the correlation of carbon-acquiring enzymes varies with the chemical nature of the available primary carbon source. Notably, a strong correlation between particulate organic carbon and *β*-glucosidase activity demonstrates their polysaccharide depolymerization in providing the carbon for microbial growth. Last, we show that exoenzyme activity patterns are not necessarily correlated with prokaryotic community composition, suggesting a redundancy of exoenzyme functions among the marine microbial community and substrate availability. This study provides foundational work for linking exoenzyme function with dissolved organic substrate and downstream processes in marine systems.

**IMPORTANCE** Microbes release exoenzymes into the environment to break down complex organic matter and nutrients into simpler forms that can be assimilated and utilized, thereby addressing their cellular carbon, nitrogen, and phosphorus requirements. Despite its importance, the factors associated with the synthesis of exoenzymes are not clearly defined, especially for the marine environment. Here, we found that exoenzymes associated with nitrogen and phosphorus acquisition were strongly correlated with inorganic nutrient levels, while those associated with carbon acquisition depended on the type of organic carbon available. We also show a linear relationship between carbon- and nitrogen-acquiring exoenzymes and a strong correlation between microbial biomass and exoenzymes, highlighting their significance to microbial productivity. Last, we show that changes in microbial community composition are not strongly associated with changes in exoenzyme activity profiles, a finding which reveals a redundancy of exoenzyme activity functions among microbial community. These findings advance our understanding of previously unknown factors associated with exoenzyme production in the marine environment.

## Abstract (precis)

Microbes release exoenzymes into the environment to break down complex organic matter and nutrients into simpler forms that can be assimilated and utilized, thereby addressing their cellular carbon, nitrogen, and phosphorus requirements. Despite its importance, the factors associated with the synthesis of exoenzymes are not clearly defined, especially for the marine environment. Here, we found that exoenzymes associated with nitrogen and phosphorus acquisition were strongly correlated with inorganic nutrient levels, while those associated with carbon acquisition depended on the type of organic carbon available. We also show a linear relationship between carbon- and nitrogen-acquiring exoenzymes and a strong correlation between microbial biomass and exoenzymes, highlighting their significance to microbial productivity. Last, we show that changes in microbial community composition are not strongly associated with changes in exoenzyme activity profiles, a finding which reveals a redundancy of exoenzyme activity functions among microbial community. These findings advance our understanding of previously unknown factors associated with exoenzyme production in the marine environment.

## INTRODUCTION

Microbial activities are a function of their specific environmental conditions, where available nutrients and cellular metabolic energy strictly regulate response. Exoenzymes are enzymes secreted by microbes to help catalyze the breakdown of high-molecular-weight polymers in the environment into simpler forms that can then be easily assimilated and utilized (1). Exoenzymes can provide essential nutrients to the microbes: for example, *β*-glucosidase can provide carbon through the breakdown of polysaccharides and similarly leucine aminopeptidase (LAP) can provide nitrogen through protein breakdown. However, exoenzymes are often higher in molecular weight than the maximum that can diffuse through the cell membrane (600 Da) (2), and their production and secretion are expensive, requiring valuable energy and cellular resources (3, 4). In addition, production and secretion of enzymes into the environment without the guaranteed return of investment are contradictory to the tightly regulated bioenergetic processes that occur intracellularly. However, the production of enzymes may be more economically efficient than previously thought. Smith and Chapman (5) demonstrated that extracellular proteins including exoenzymes exhibit economical and resource selection with these proteins costing 1.3 fewer ATPs; lower carbon, nitrogen, and sulfur contents; and less Gibbs free energy than cytoplasmic proteins. For example, extracellular serine proteases were less expensive by 0.72 ATPs than their intracellular counterparts.

Extracellular secretion of enzymes by microbes is observed across all habitats (i.e., marine, freshwater, and terrestrial). Factors regulating exoenzyme production, activity, and their stability vary depending on the system (6). Sinsabaugh et al. (7) showed that in soil and sediments, the production of exoenzymes is regulated by nutrient availability. Factors such as pH and temperature are less significant in marine environments, and hot spots of microbial activity (e.g., marine snow) dominate the synthesis and excretion of enzymes (6, 8–10). However, the relationship between regulating factors and exoenzymes remained to be defined for our oceans (3, 6). This is primarily due to their vast spatial-temporal scales, the largely uncharacterized nature of dissolved and particulate organic matter (11), and methodological limitations. The objective of this study was to determine factors that trigger the production of exoenzymes in the marine water column using mesocosms as a model system. As an extension of this objective, we also determine the significance of exoenzyme activities to microbial biomass and how their activities respond to factors such as available nutrients and high-molecular-weight organic substrates.

Several studies have shown a strong relationship between the composition of bioavailable organic substrates and microbial communities (12–15). As exoenzymes are one of the major toolkits microbes use for high-molecular-weight substrate degradation and resource acquisition, we predict that there will be a strong link between the enzyme activity patterns, chemical composition of the bioavailable substrates, and microbial community composition. We hypothesize that changes in these parameters will be parallel, with processes continuously influencing each other through time. This is true for terrestrial (16, 17) and freshwater aquatic (18) systems, but the exact mechanism of such correlations for marine ecosystems remains to be established (12–15).

Oil is a complex mixture of aliphatic and aromatic hydrocarbons that can undergo a series of transformations in its chemical characteristics through action of microbes and natural weathering (19, 20), making it ideal to study the interplay of changing substrate characteristics, microbial community composition, and exoenzyme activities. Therefore, we used naturally occurring organic carbons and oil as available substrates to test the link between changes in substrate, exoenzyme activities, and microbial community composition through time. To do this, we conducted a mesocosm experiment with a control (no treatment) and a water-accommodated fraction (WAF) of oil treatment. The control consisted of seawater from the Gulf of Mexico, where natural polysaccharides, and to a lesser extent proteins, produced predominantly by phytoplankton (21), are the dominant form of total organic matter and hence the primary carbon source. In the WAF treatment, oil acts as the primary carbon source until it is depleted, after which natural polysaccharides may become important. The mesocosm experiment was run for 16 days, during which activities of the exoenzymes *β*-glucosidase, LAP, lipase, and alkaline phosphatase (AP), along with nutrient concentration, organic matter composition and concentration, and prokaryotic community composition, were measured. Overall, we found that the exoenzyme activities are tightly correlated with factors such as nutrient concentrations and the chemical nature of organic substrates. We also found that the changes in exoenzyme activity profile did not correspond with a parallel change in microbial community composition, suggesting a redundancy in enzyme function across the microbial communities in the marine system.

## RESULTS

### Hydrocarbon concentrations.

**FIG 1:**
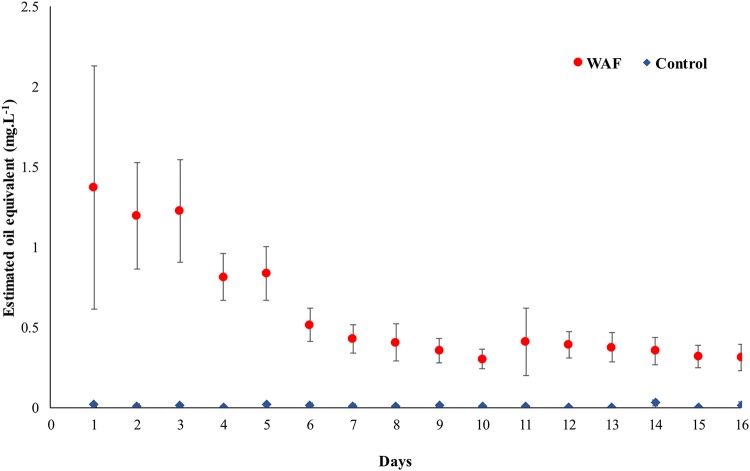
Estimated oil equivalent values (average ± standard deviation), a proxy of oil concentration in the WAF system monitored every day throughout the course of the experiment (*n* = 3, for each day).

The initial concentration of oil was 1.37 (±0.76) mg·liter^−1^ in the WAF mesocosm tanks. We observed a continuous decrease in the estimated oil equivalent values with time during the first 7 days of the experiment (Fig. 1). No significant changes in estimated oil equivalents values were observed thereafter. Oil concentrations were low but above the detection limits beyond this point.

### Enzyme activities versus nutrient concentrations.

**FIG 2:**
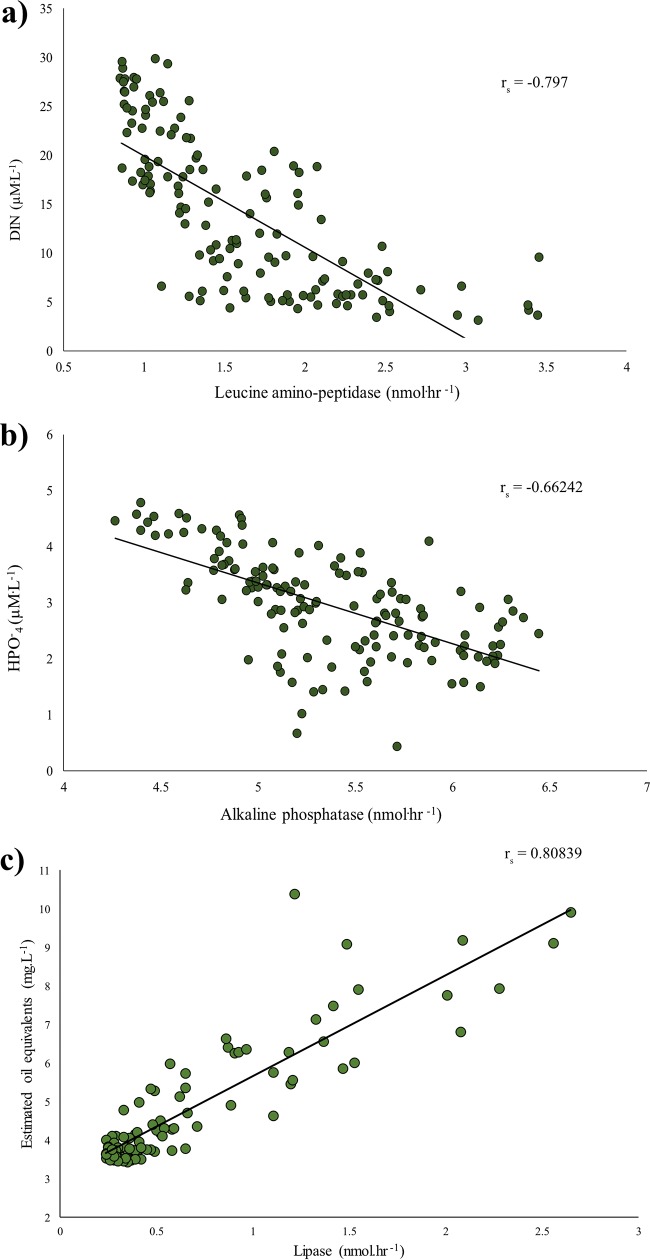
(a) Correlation plots between leucine aminopeptidase activities (nmol·h^−1^) and dissolved inorganic nitrogen concentration (μM·liter^−1^) (*n* = 134). (b) Alkaline phosphatase activities (nmol·h^−1^) and inorganic phosphate concentration (μM·liter^−1^) (*n* = 142). (c) Lipase activities (nmol·h^−1^) and estimated oil equivalent (*n* = 97).

Initial dissolved inorganic nitrogen (DIN) and dissolved inorganic phosphate (DIP) concentrations after the nutrient amendment in the control tanks were 29.54 (±6.94) μM·liter^−1^ and 4.46 (±0.24) μM·liter^−1^, respectively, while in the WAF tanks they were 26.57 (±1.19) μM·liter^−1^ and 4.41 (±0.13) μM·liter^−1^, respectively. To determine whether nutrient concentrations influenced the enzyme production in the mesocosms, the enzyme activities from both treatments (see Fig. S1a to d in the supplemental material) were compared against the corresponding nutrient concentrations at the same time point (Fig. 2; see also Fig. S1e and f). We found the activities of LAP were inversely correlated with the dissolved inorganic nitrogen concentrations (Spearman’s rho value [*r_s_*] = −0.797, *P* < 0.0001) (Fig. 2a). Similarly, we found that the activities of AP were inversely correlated with the corresponding dissolved inorganic phosphate concentrations in the water samples (*r_s_* = −0.662, *P* < 0.0001) (Fig. 2b). When the activities of the oil-degrading enzyme lipase (22–24) were compared to the total oil concentrations, we found a significant linear correlation between these two parameters (*r_s_* = 0.808, *P* < 0.0001) (Fig. 2c).

10.1128/mSystems.00290-20.1 FIG S1 Exoenzyme activities. (a) *β*-Glucosidase activities (nmol·h^−1^). (b) Lipase (nmol·h^−1^). (c) Leucine aminopeptidase activities (nmol·h^−1^). (d) Alkaline phosphatase (nmol·h^−1^) and nutrient concentrations of DIN (μmol·liter^−1^) and DIP (μmol·liter^−1^) versus time in the control and WAF treatment during the entire course of the experiment. Download FIG S1, TIF file, 1.4 MB. Copyright © 2020 Kamalanathan et al. 2020 Kamalanathan et al. This content is distributed under the terms of the Creative Commons Attribution 4.0 International license.

### Correlations between activities of different exoenzymes.

**FIG 3:**
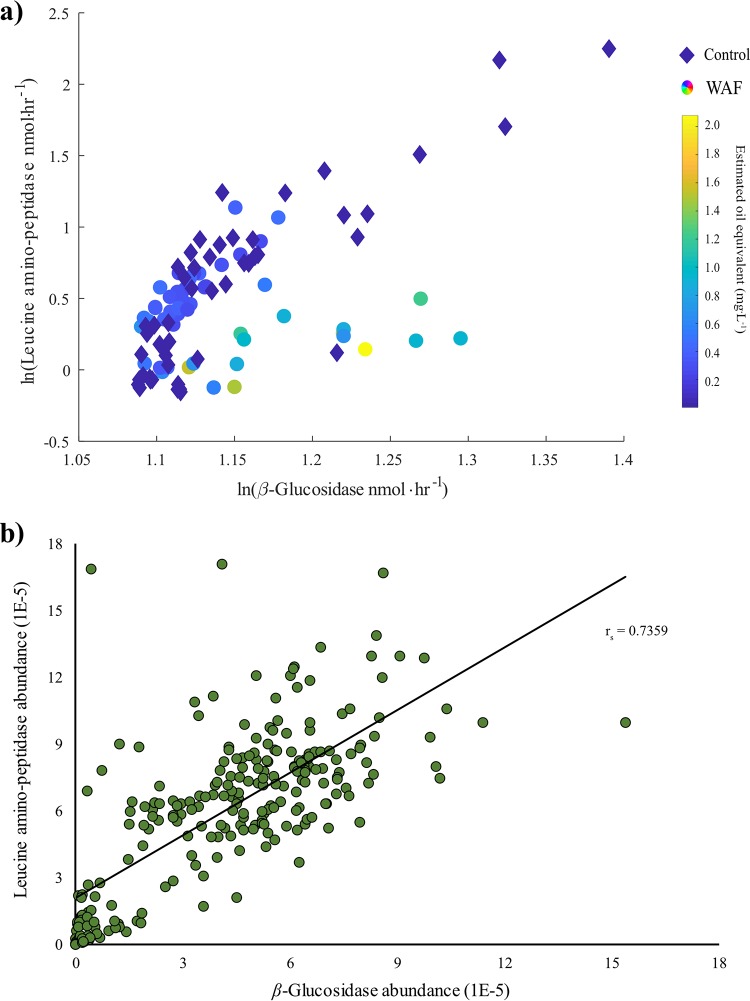
Correlation plots between (a) β-glucosidase activities (nmol·h^−1^) and leucine aminopeptidase activities (nmol·h^−1^) (*n* = 48) in the control and WAF system during the entire course of the experiment. (b) Homolog abundance of *β*-glucosidase homologs and leucine aminopeptidase in the TARA Oceans data set (*n* = 242).

We compared activities of the carbon acquisition enzyme *β*-glucosidase (which provides carbon through breakdown of polysaccharides) with the nitrogen acquisition enzyme LAP (which provides nitrogen through protein breakdown) under control and WAF conditions to determine if substrate availability governs this relationship. We found that under control conditions, *β*-glucosidase activities were linearly correlated with LAP activities throughout the course of the experiment (*r_s_* = 0.852, *P* < 0.0001) (Fig. 3a). Supportive of this linear relationship in marine systems, a linear correlation was observed between the global homolog abundance of *β*-glucosidase and LAP derived from the global sampling project TARA Ocean Database (*r_s_* = 0.7359, *P* < 0.0001) (Fig. 3b), indicating that a linear relationship in both activity rates (mesocosms) and potential for gene production (TARA metagenomes) is the rule in marine systems. However, such a correlation between *β*-glucosidase and LAP was not observed for the complete duration of the experiment in the WAF treatment (Fig. 3a and Fig. S2). Spearman’s rho versus time and oil concentrations revealed a dual phase in the correlation between these two enzymes in the WAF treatment (Fig. S3), with relatively weaker correlation between *β*-glucosidase and LAP during the first 7-day period (*r_s_* = 0.65974, *P* = 0.00114), which is also the period when oil was present at higher concentration. This was followed by a significant positive correlation (*r_s_* = 0.857, *P* < 0.0001) during the next 9 days, during which time the oil concentration was consistently low (Fig. 3a and Fig. S2 and S3).

10.1128/mSystems.00290-20.2 FIG S2 Correlation plots between *β*-glucosidase activities (nmol·h^−1^) and leucine aminopeptidase activities (nmol·h^−1^) (*n* = 47) in the WAF treatment during the entire course of the experiment. Download FIG S2, TIF file, 0.9 MB. Copyright © 2020 Kamalanathan et al. 2020 Kamalanathan et al. This content is distributed under the terms of the Creative Commons Attribution 4.0 International license.

10.1128/mSystems.00290-20.3 FIG S3 Spearman’s rho (*r_s_*) plotted against various time intervals (bottom *x* axis) for β-glucosidase versus leucine aminopeptidase (blue diamonds, left *y* axis) and lipase versus leucine aminopeptidase (red circles, right *y* axis). The significance of the correlation is represented by the size of the data points of Spearman’s rho (*r_s_*). The plot also shows estimated oil equivalents (black squares, left *y* axis) plotted against time (top *x* axis). Download FIG S3, PDF file, 0.08 MB. Copyright © 2020 Kamalanathan et al. 2020 Kamalanathan et al. This content is distributed under the terms of the Creative Commons Attribution 4.0 International license.

**FIG 4:**
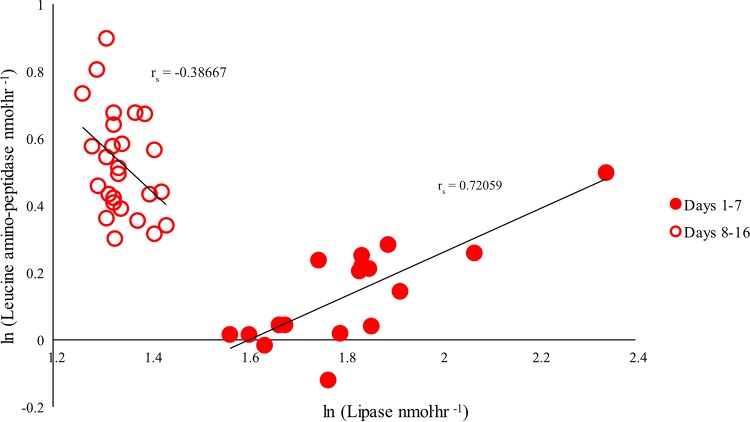
Correlation plots between lipase activities (nmol·h^−1^) and leucine aminopeptidase activities (nmol·h^−1^) (*n* = 43) in the WAF system during the entire course of the experiment.

We further determined if the activities of the enzyme acquiring carbon through oil breakdown (lipase) and the nitrogen acquisition enzyme LAP are correlated under control and WAF conditions. No correlation was observed for the complete duration of the experiment between lipase and LAP under control conditions (*r_s_* = −0.42105, *P* = 0.06449, for days 1 to 7 and *r_s_* = 0.3306, *P* = 0.08574, for days 8 to 16) (Fig. S4). In the WAF treatment, a dual-phase correlation was observed; in the first phase, lipase positively correlated with LAP (*r_s_* = 0.72059, *P* = 0.0011) while a weak correlation between *β*-glucosidase and LAP was observed (Fig. 4; see also Fig. S2 and S3). This was followed by absence of correlation between lipase and LAP (*r_s_* = −0.38667, *P* = 0.05102) when *β*-glucosidase positively correlated with LAP during the second phase (Fig. 4; see also Fig. S2 and S3).

10.1128/mSystems.00290-20.4 FIG S4 Correlation plot between lipase (nmol·h^−1^) and leucine aminopeptidase (nmol·h^−1^) (*n* = 48) in the control treatment during the entire course of the experiment. Download FIG S4, PDF file, 0.06 MB. Copyright © 2020 Kamalanathan et al. 2020 Kamalanathan et al. This content is distributed under the terms of the Creative Commons Attribution 4.0 International license.

### Correlations between POC concentrations against colloidal organic matter concentrations and enzyme activities.

Next, we determined how exoenzymes aided acquisition of nutrient-influenced microbial biomass by assuming particulate organic carbon (POC) as a proxy of microbial biomass. We tested the correlation of POC with factors such as exoenzymes (individual and total activities), protein and polysaccharide content of the exopolymeric substance (EPS), organic carbon, and nitrogen content of the colloidal fraction of the water samples from all the treatments. Multiple linear regression revealed significant positive correlations between POC and *β*-glucosidase (*r_s_* = 0.6556, coefficient = 1.4587, *P* = 3.47E−07); however, other parameters did not show any correlations (Table 1). In addition, we also used correlation matrix analysis to further test the correlation between POC and the above-mentioned factors. Similarly to multiple linear regression, POC showed a significant linear correlation with *β*-glucosidase (*r_s_* = 0.77, *P* < 0.0001) and a weaker correlation for LAP (*r_s_* = 0.3, *P* = 0.04), but not for other variables (Table S1).

**TABLE 1:** Summary statistics for multiple linear regression for POC against β-glucosidase, LAP, lipase, AP, total enzyme activities, uronic acids, neutral sugars, proteins, total EPS COC, and CON

Component	Coefficient	SE	*t* value	Pr(>|*t*|)	*df*	*F* value
(Intercept)	−3.09899	0.631623	−4.906	1.88E−05		
Glucosidase	1.458743	0.235657	6.19	3.47E−07	38.3174	3.47E−07
Peptidase	−0.048	0.058534	−0.82	0.4175	0.6724	0.41748
Lipase	−0.06669	0.046456	−1.436	0.1595	2.0607	0.15954
Phosphatase	−0.12812	0.070564	−1.816	0.0775	3.2964	0.07754
Uronic acids	2.958485	1.714474	1.726	0.0928	2.9777	0.09276
Neutral sugars	0.11159	0.320795	0.348	0.7299	0.121	0.72992
Proteins	0.456929	1.299779	0.352	0.7272	0.1236	0.72717
COC	−0.00436	0.78728	−0.006	0.9956	0	0.99561
CON	−7.7633	8.06278	−0.963	0.3419	0.9271	0.34187

10.1128/mSystems.00290-20.6 TABLE S1 Summary statistics of correlation matrix analysis for POC, β-glucosidase, leucine aminopeptidase (LAP), lipase, alkaline phosphatase (AP), total enzyme activities, uronic acids, neutral sugars, proteins, total EPS colloidal organic carbon (COC), and colloidal organic nitrogen (CON). Download Table S1, DOCX file, 0.01 MB. Copyright © 2020 Kamalanathan et al. 2020 Kamalanathan et al. This content is distributed under the terms of the Creative Commons Attribution 4.0 International license.

### Bacterial community composition and exoenzyme profile.

**FIG 5:**
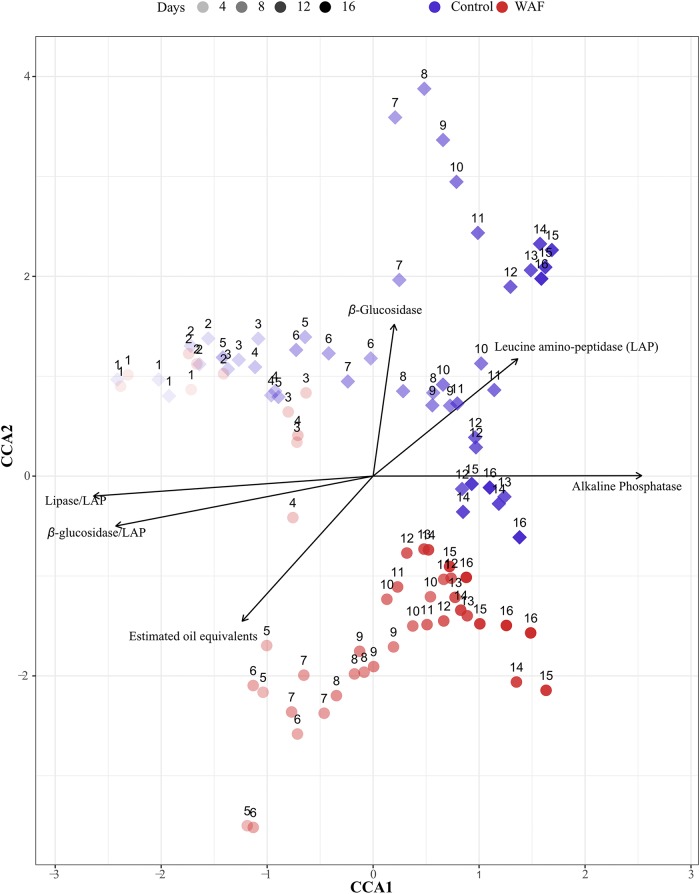
CCA plot comparing the microbial community structure (filled circles) with environmental variables (arrows) in the control (blue diamonds) and WAF (red circles) system throughout the course of the experiment. Only the significantly correlated environmental variables are displayed on the plot.

The starting microbial community compositions in the WAF and control tanks were similar but diverged over the course of the experiment (Fig. 5). Canonical correspondence analysis (CCA) was performed to identify the relationship between environmental variables such as the microbial community structure, DIN, DIP, estimated oil equivalents, exoenzyme (*β*-glucosidase, lipase, LAP, and AP) activities, and ratios of carbon and nitrogen acquisition (*β*-glucosidase/LAP, lipase/LAP) (Fig. 5). Ratios of these enzymes were included in the analysis as relationships between carbon- and nitrogen-acquiring enzyme correlation with oil concentration and the two time periods (Fig. 3 and 4; see also Fig. S2 and S3). Parameters associated with EPS and organic carbon were excluded due to gaps in time course measurements. Ordistep and analysis of variance (ANOVA) using the CCA model suggested that DIN and DIP were insignificantly related to the microbial community structure (ANOVA; *P* = 0.159 for DIN and *P* = 0.393 for DIP). Reanalysis of environmental variables with the microbial community composition by excluding DIN and DIP explained 40% of the variation. All of the environmental variables were significantly related with the microbial community structure, with the exception of lipase. Permutation tests revealed estimated oil equivalents and lipase/LAP to be the most significantly correlated environmental parameter (permutation test: estimated oil equivalents, *F* = 10.7235, *P* = 0.0002; lipase/LAP, *F* = 24.4338, *P* = 0.0002). LAP, *β*-glucosidase/LAP, AP, and *β*-glucosidase also showed significant correlations with the microbial community as well (permutation test: LAP, *F* = 6.0807, *P* = 0.0002; *β*-glucosidase/LAP, *F* = 3.7192, *P* = 0.0014; AP, *F* = 6.8659, *P* = 0.0002; *β*-glucosidase, *F* = 5.7127, *P* = 0.0002). The orthogonal projections (Fig. 5) suggest that *β*-glucosidase and LAP are correlated with the control community structure. On the other hand, estimated oil equivalents were strongly associated with WAF community structure, projecting oppositely from the control communities and *β*-glucosidase and LAP vectors, especially during the time point days 5 to 16. The vector lengths of environmental variables such as *β*-glucosidase/LAP, lipase/LAP, and alkaline phosphatase were indicative of the higher strength in association between these variables and microbial community structure.

Analysis of microbial community composition showed that members belonging to the order *Alteromonadales* and *Oceanospirillales* persisted longer in the WAF treatment than the control (Fig. S5). Members of the *Methylococcales* were observed only in the WAF, appearing to peak at day 5 before subsequently declining (Fig. S5). Unclassified *Betaproteobacteriales* amplicon sequence variant (ASVs) increased in abundance following the start of the experiment in both the control and WAF tanks. Similarly, members of the order *Cellvibrionales* appeared during the later phases (day 8 onward) of the experiment in both controls and WAF, with relatively higher abundance in the WAF (Fig. S5). Members of the order *Chitinophagales* that were absent in the WAF appeared in the control from day 12 onward (Fig. S5).

10.1128/mSystems.00290-20.5 FIG S5 Microbial community composition in the control and WAF treatments throughout the course of the experiment. Only orders representative of >3.5% of the total community are presented. Download FIG S5, PDF file, 0.01 MB. Copyright © 2020 Kamalanathan et al. 2020 Kamalanathan et al. This content is distributed under the terms of the Creative Commons Attribution 4.0 International license.

## DISCUSSION

Microbes, especially bacteria, acquire nutrients from the environment by hydrolyzing complex organic matter with the help of exoenzymes. Although factors regulating their production and correlation with the microbial community are known for other ecosystems (6, 7), little is known for the marine biome. In this study, we attempt to address these concerns with the help of a mesocosm study.

### Factors regulating exoenzyme production in a marine system.

In the past, a correlation between polysaccharide-degrading *β*-glucosidase and protein-degrading LAP was used as an indicator of relative importance of polysaccharide- or protein-based metabolism (22, 23, 25). In accordance with these studies, we observed a strong correlation between *β*-glucosidase and LAP in our controls. In addition, we found that the global abundance *β*-glucosidase homologs correlated strongly with that of LAP, highlighting the global strength of this correlation and the balance between carbon and nitrogen acquisition in the marine environment. However, in the WAF, we found a weak correlation between *β*-glucosidase and LAP during the first 7 days, which became stronger thereafter. Although, as hypothesized in previous studies (22, 23, 25), one could expect a shift between polysaccharide/protein-based systems, correlation plots between lipase and LAP reveal a new insight into this process. Lipase activities strongly correlated with LAP activities during the first 7 days when no strong correlation between *β*-glucosidase and LAP was observed. This correlation switched from between lipase and LAP to *β*-glucosidase and LAP from day 8 onward and coincided with the near-depletion of oil levels in this system. This suggests that oil acted as a primary carbon source during the first 7 days, followed by polysaccharides acting as carbon source thereafter. The absence of any correlation between lipase activities and LAP and a strong positive correlation between *β*-glucosidase and LAP in the control treatment suggests that polysaccharides acted as the primary carbon source throughout the course of the experiment for that microbial community.

Overall, these observations highlight three important findings. (i) The *β*-glucosidase/LAP ratio has been used previously as an indicator of seasonal variation of bacterioplankton substrate utilization, with higher ratios indicative of dominant use of polysaccharides and lower ratios indicative of dominant use of proteins (22, 23, 25). However, Fukuda et al. (22) show that while these ratios are a good indicator of bacterial utilization of organic polymers, factors like zinc concentration can affect that trend. Our study suggests that changes in ratio between *β*-glucosidase and LAP activities may also reflect the presence of an additional carbon substrate chemically different from the *β*-glucosidase substrate polysaccharide (oil in our study). (ii) The strong correlation between lipase activities and the oil concentration suggests that the expression and production of this enzyme are dependent on the substrate concentration. (iii) The switch from a strong correlation between lipase and LAP to one between *β*-glucosidase and LAP until the near-depletion of oil suggests that the primary carbon source in a given system tightly regulates the type of carbon acquisition enzymes expressed. Clinton et al. (25) noted a similar change, with a strong correlation between *β*-glucosidase and LAP which changed when the dissolved organic carbon (DOC) composition of the river changed. In addition, our observation of a rapid change in correlation from lipase and LAP to *β*-glucosidase and LAP also indicates a relatively shorter half-life of lipase.

The negative correlation between activities of LAP and DIN suggested that this enzyme was induced in response to nitrogen limitation. Such an inverse correlation has been observed previously (24, 26, 27). However, the correlation between carbon-acquiring enzyme *β*-glucosidase and LAP suggests carbon acquisition as an additional regulating factor, in addition to inorganic nitrogen concentration. Although LAP has been shown to play a role in carbon acquisition as well (26–28), its linear relationship with carbon acquisition enzymes *β*-glucosidase and lipase suggest that they were primarily induced to acquire nitrogen and not carbon in our experiment. We hypothesize that the amino acids produced through enzymatic action of peptidase are acquired and assimilated directly into protein molecules, instead of deamination/transamination of the product to derive carbon.

The observed increase in AP activity with decreasing orthophosphate concentrations is consistent with the findings reported elsewhere (29, 30), including in the Gulf of Mexico, wherein an inverse correlation between AP activity and orthophosphate concentrations was also demonstrated (31). Overall, our data suggest that the production of these enzymes and the levels of their activities are influenced by nutrient levels and the kind of carbon substrate available. However, effects of other environmental factors such as temperature, pH, and surface area on exoenzyme activity in the marine environment require further investigation.

### Enzyme activities and microbial productivity.

It is well known that dissolved organic matter secreted by phytoplankton, either actively as EPS or passively by cell death or phage lysis, is a major source of organic carbon and nitrogen substrates for heterotrophic bacteria (32, 33). Nutrient acquisition has been shown to have a direct relationship with heterotrophic biomass production in river, soil, and sediment systems (7, 34), as assimilable products generated by enzymatic depolymerization reactions can be directly be taken up to meet their carbon, nitrogen, and phosphorus demands. Such reactions have been shown to be the rate-limiting step for bacterial assimilation of macronutrients and therefore can regulate bacterial productivity (34). We observe a strong correlation between *β*-glucosidase and POC concentration. According to the work of Chróst (35), extracellular enzymes (cell free) consist of only 8 to 15% of the activities, with cell-bound/ectoenzyme accounting for the remainder. Previous studies have shown good correlation between bacterial production and POC (36, 37). Assuming POC content as a proxy of biomass, the strong positive correlation between *β*-glucosidase activities and POC concentration implicates carbon from polysaccharide depolymerization reactions as a critical factor for microbial growth in our mesocosms. These results suggest that polysaccharides in EPS may be depolymerized by *β*-glucosidase, contributing to biomass production and serving as a link between DOC and POC.

### Enzyme activities and microbial communities.

The diverging nature of the microbial community composition with time between the two treatments clearly highlights the influence of the initial presence of a different carbon substrate (in our case, oil) on the fate of the progression of community composition. The microbial community composition in the control is associated with *β*-glucosidase and LAP, as indicated by the CCA plot and by the continual presence of members of the order *Betaproteobacteriales*, and the appearance of members of the orders *Cellvibrionales* and *Chitinophagales* in the latter phase. Members belonging to these orders are over 53% positive for *β*-glucosidase based on IMG database search (38). The microbial community composition in the WAF treatments showed trends that are classically associated with the presence of oil, indicated from the stronger association between estimated oil equivalents and WAF community composition in the CCA. This was further confirmed by the relatively extended presence of members belonging to the order *Alteromonadales* compared to control and the higher abundance of *Oceanospirillales* members compared to the control system. Both these orders have bacteria that are known hydrocarbon degraders and lipase producers (39–41). In addition, members of the *Methylococcales* order, which are likely methane oxidizers and/or lipase producers, were also observed in the WAF (42–44). However, members of the *Methylococcales* order occurred primarily during the latter part (days 5 to 8) of the first phase (days 1 to 7) of the WAF treatment, and around 93% of sequences classified in this order were classified as *Cycloclasticus* spp. (data not shown), which are known aromatic hydrocarbon degraders (45) and lipase producers (46, 47). The occurrence of members of the order *Betaproteobacteriales* in the WAF during the latter phase coincided around the time of switch in major carbon substrate utilization from oil to polysaccharides. This observation, along with their almost continual presence in the controls, highlights the influence of the nature of carbon substrate on the *Betaproteobacteriales* members that were present in our two mesocosm treatments. Similarly to the control, members of the orders *Cellvibrionales* and *Chitinophagales* were observed during the latter phase in WAF. Their sustained abundance until the end of the experiment suggests they might be utilizing polysaccharides through *β*-glucosidase.

From the observed trend in enzyme activity, i.e., a correlation between *β*-glucosidase and LAP, one could expect a stronger link between the enzyme activity pattern and microbial community composition, in particular, a similarity between the community composition in the WAF treatment and that observed in the controls during the latter part of the experiment. However, the observation of the contrary pattern in the microbial community composition suggests that similar enzyme activity patterns are not necessarily a reflection of a similar microbial community structure, despite the strong correlation between the two parameters and the ability of the enzyme activity pattern to predict the environmental conditions under which they were produced. We assume this is primarily due to the redundancy of the enzyme activity functions among the different microbes. This relatively conserved nature of exoenzymes that can aid in the nutrient acquisition processes regardless of the microbial community composition highlights the important nature of this process in nutrient cycling in the marine environment.

In conclusion, we present here evidence that exoenzyme activities in marine systems are regulated by the type of organic carbon present. Supporting previous studies, we found a relationship between *β*-glucosidase and LAP, and this relationship was supported by genomic content analyzed during the TARA global sampling expedition. However, the addition of oil disrupted the linear relationship between *β*-glucosidase and LAP, and instead, lipase activity was linear with LAP while oil was present in high-enough concentrations to be the primary carbon source for the bulk of the community and then shifted back to the canonical relationship when it was exhausted. Interestingly, such changes in linear relationship between carbon- and nitrogen-acquiring enzymes were not mirrored in the prokaryotic community composition, which indicates that functional redundancy for exoenzyme production exists, or that shifts in composition are decoupled from activity rates for the exoenzymes measured here.

## MATERIALS AND METHODS

### Mesocosm setup.

Mesoscale (87 liters; tanks were 74.5 cm long by 43cm wide) experiments were conducted using seawater collected from the Gulf of Mexico (29.2726N, 94.8126W; salinity, 25; pH 7.9; temperature, 30.8°C) on 23 May 2017. The seawater was supplemented with nutrients at f/20 concentrations (48). Controls (3 tanks) comprised just seawater. Treatment tanks (3 tanks) were amended with WAF produced according to the method of Wade et al. (49) using Macondo surrogate oil. Briefly, the oil (25 ml) was added to seawater in a 130-liter circulating baffled tank and mixed for 4 h at ambient temperature (∼21°C) under low light to avoid photooxidation. The WAF was then transferred to the tanks, avoiding the surface slick. Mesocosm tanks were incubated at ambient temperature (∼21°C), an average light intensity of 60 μmol photons m^−2^ s^−1^, and a 12:12 day/night cycle for 16 days.

### Oil analysis.

Estimated oil equivalents were measured every 24 h according to the method of Wade et al. (50) from a spigot 10 cm above the bottom of each tank. Briefly, the fluorescence of dichloromethane extracts (5 to 10 ml) was measured at 260/358-nm excitation/emission, respectively, using a spectrofluorometer (Shimadzu RF-5300). The florescent response was compared to a five-point calibration curve prepared using Macondo surrogate oil.

### Exoenzyme assays.

The activities of exoenzymes *β*-glucosidase, LAP, AP, and lipase were measured daily for 16 days according to methods described by Kamalanathan et al. (51). Substrates 4-methylumbelliferyl-β-d-glucopyranoside, 4-methylumbelliferyl-oleate, 4-methylumbelliferyl-phosphate, and leucine-amidomethylcoumarin (AMC) hydrochloride were used for activities of enzymes β-glucosidase, lipase, AP, and LAP, respectively. The samples were incubated with respective substrates at 0.2 mM (final concentration) for 3 h, and activities were determined as fluorescence at excitation/emission wavelengths of 365/448 nm for methylumbelliferyl-tagged substrates and 380/440 nm for AMC-tagged substrates, respectively. Measurements were performed using a BioTek Cytation 5 imaging reader controlled by Gen5 2.09 software (BioTek, USA).

### Data mining for *β*-glucosidase and LAP.

Global homolog abundances of *β*-glucosidase and LAP were derived from the TARA Ocean Database by querying the protein sequence of EC 3.2.1.21 (*β*-glucosidase) and EC 3.4.11.1 (LAP) in the Ocean Gene Atlas webserver (http://tara-oceans.mio.osupytheas.fr/ocean-gene-atlas/) (52, 53).

### Nutrient analysis.

Dissolved inorganic nitrogen (DIN as the sum of NO_2_^−^, NO_3_^−^, and NH_4_^+^) and dissolved inorganic phosphate (DIP) were determined by filtering 30-ml samples from each set of treatment tanks (*n* = 3) through a 45-μm-diameter glass microfiber filter (Millipore) and freezing the filtrate (−20°C). Samples were analyzed with an Astoria Pacifica autoanalyzer with a Certified Reference Material (CRM). Flow Analyzer software package II (FASPACII) was used to analyze peak height and its conversion to micromolar·liter^−1^ concentrations.

### POC and nitrogen PON analysis.

Particulate organic carbon (POC) and particulate organic nitrogen (PON) analyses were performed by filtering water samples (*n* = 3) through a precombusted GF/F membrane (0.7 μm; Whatman, USA). After removing the carbonates by HCl fuming, quantification was performed on a Perkin-Elmer Series II CHNS 2400 analyzer with acetanilide (71.09%) as the analytical standard (54).

### COC and CON analysis.

For colloidal organic carbon (COC) and colloidal organic nitrogen (CON) measurements, ∼150-ml samples from each treatment were collected and filtered through a Flipmate 100 filtration system (0.4-μm polyethersulfone; Environmental Express, USA). The aliquots of the filtrate (<0.4 μm) were further ultrafiltered through an Amicon Ultra-15 centrifugal filter unit with a 3-kDa-cutoff membrane (Millipore, USA). The retentate (3 kDa, 0.4 μm) was then diafiltered with ultrapure water (18.2 MΩ) and concentrated to 2 ml for all further analysis. COC and CON were measured with a Shimadzu TOC-L analyzer according to the method of Xu et al. (55).

### EPS analysis.

Exopolymeric substance (EPS) was determined as the sum of neutral sugars, protein, and uronic acids in 0.4-μm retentate. Water samples were filtered through polycarbonate filters (0.4 μm, Millipore) to collect particles and extract the attached EPS (with 0.35 M EDTA). This was followed by an ultrafiltration step to aid the removal of excessive EDTA and salts (56). For extracting EPS from the dissolved phase, an Amicon Ultra-15 centrifugal filter unit with an Ultracel-3 membrane (Millipore; 3 kDa) was used for concentrating and desalting purposes. Both EPS fractions were pooled, and the carbohydrate concentration was determined by the anthrone method with glucose as the standard (57). The Pierce bicinchoninic acid (BCA) protein assay kit based on a modified bicinchoninic acid method with bovine serum albumin as the standard was used for the estimation of protein content of EPS (57). Uronic acids in the EPS were determined according to the method of Hung and Santschi (58) and Filisetti-Cozzi and Carpita (59). Distilled water was used as an analytical blank for determination of neutral sugars, protein, and uronic acids.

### Bacterial community composition.

Sequencing of 16S rRNA gene amplicons was performed as described in the work of Doyle et al. (39). Briefly, 250 ml of water from each tank (*n* = 3) was vacuum filtered (≤20-cm Hg) through a 47-mm, 0.22-μm Supor PES filter membrane (Pall; Port Washington, NY) daily to concentrate microbial cells following an initial prefiltration through a 10-μm filter to remove most eukaryotic cells. The filters were then stored at −80°C. Total DNA was extracted from filters using FastDNA Spin kits (MP Biomedical, Santa Ana, CA) and stored at −20°C. A 515F-806R primer pair as described in the work of Walters et al. (60), modified with barcodes and Illumina adapters, was used to amplify all samples in triplicate 25-μl PCR mixtures. Amplicons were quantified with the QuantiFluor dsDNA system (Promega), pooled at equimolar concentrations, and purified with an E.Z.N.A Cycle-Pure PCR purification kit (Omega Bio-Tek, Norcross, GA). The purified library was sequenced on the Illumina MiSeq platform (v2 chemistry, 2 by 250 bp) at the Georgia Genomics Facility (Athens, GA). Sequence read curation and processing were carried out using DADA2 (61) with the following filtering parameters: maxN = 0, truncQ = 2, rm.phix = TRUE, maxEE = 2, R1 truncLen = 240, R2 truncLen = 200. Error rates for the filtered and trimmed R1 and R2 reads were calculated using the learnErrors function and subsequently used to denoise reads using the DADA2 sample inference algorithm. The denoised reads were merged together into amplicon sequence variants (ASVs) using a global end-free alignment. Paired reads containing any mismatches in the overlapping region were removed from the data set. Chimeric ASVs were identified and removed by using the consensus method within the removeBimeraDenovo function. As a final curation step, any ASVs of which ≥0.1% of their reads were from a protocol blank sample were removed. A consensus taxonomy for each ASV was then assigned using the naive Bayesian classified method of reference 62 trained on release 132 of the SILVA reference database (63).

### Statistical analysis.

Regression analysis for the enzyme activities versus nutrient analysis and pairwise enzyme comparisons were performed using GraphPad Prism software (version 7.04). A Kolmogorov-Smirnov test showed that the exoenzyme activity data were not normally distributed; therefore, Spearman’s rho values (*r_s_*) and the significance of these correlations were determined. *r_s_* values for different time intervals (forward elimination of days) were also calculated. Examination of patterns in microbial community structure was performed using canonical correspondence analysis (CCA). CCA was also used to test the associations between microbial communities and environmental variables (64, 65). CCA was performed in R software using the vegan package (66). Both multiple linear regression and correlation matrix analysis of various factors versus POC were performed in R software using the vegan package (66). Multiple linear regression was performed using a step function that used both removal and addition of variables, and the results were analyzed using two-way ANOVA. Correlation analysis was performed using the Spearman method, and the *P* values were corrected for multiple hypotheses using the Bonferroni correction.

### Data availability.

Data are publicly available through the Gulf of Mexico Research Initiative Information and Data Cooperative at DOIs 10.7266/n7-z8x6-km14 and 10.7266/2J7H8GGS.
