# Supplementary figures and images for: Exoenzymes as a Signature of Microbial Response to Marine Environmental Conditions
Source: mSystems. 2020 Apr 14;5(2):e00290-20. doi: 10.1128/mSystems.00290-20 (PMC7159900; doi:10.1128/mSystems.00290-20)

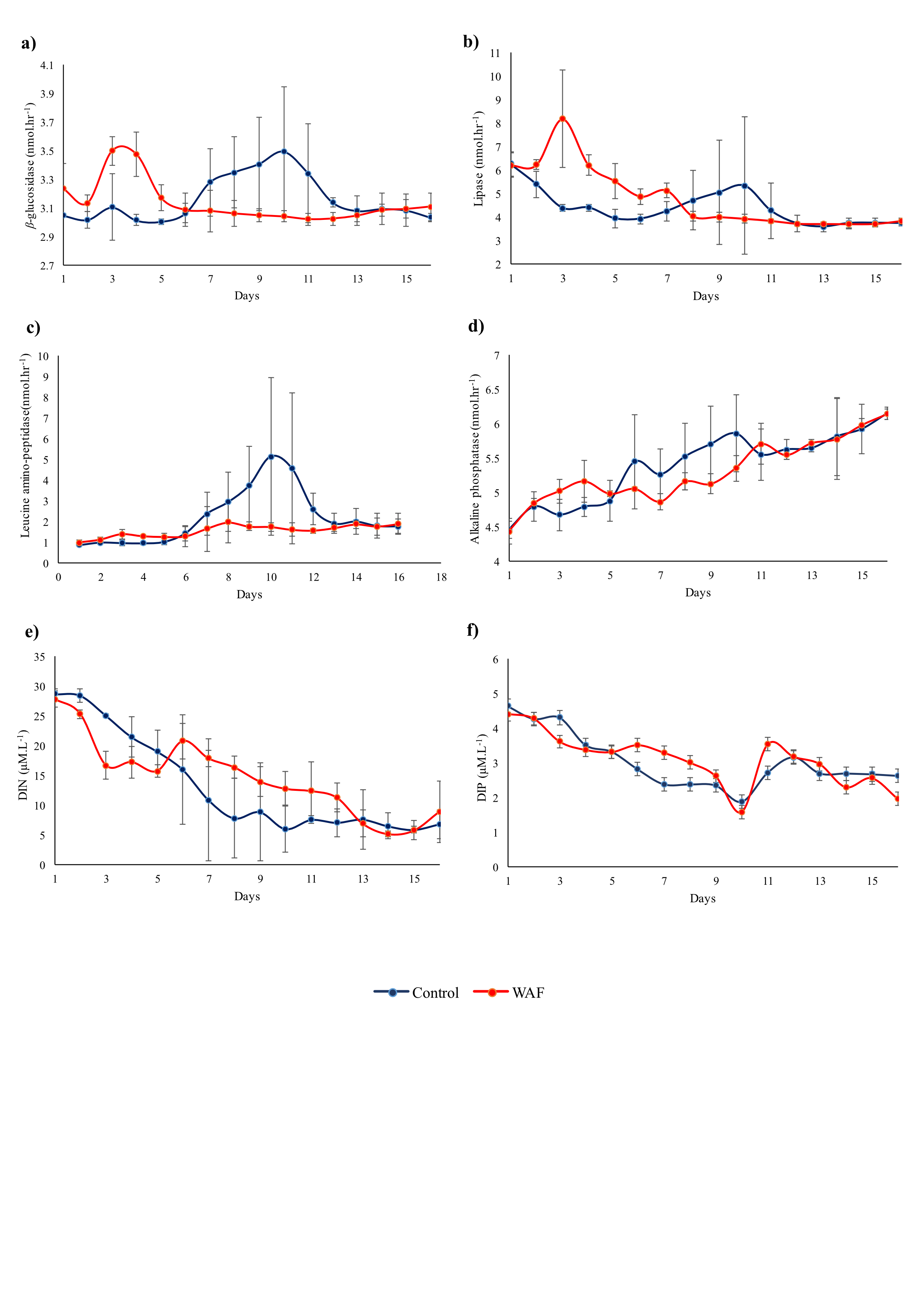

Supplement: FIG S1 [file mSystems.00290-20-sf001.tif]

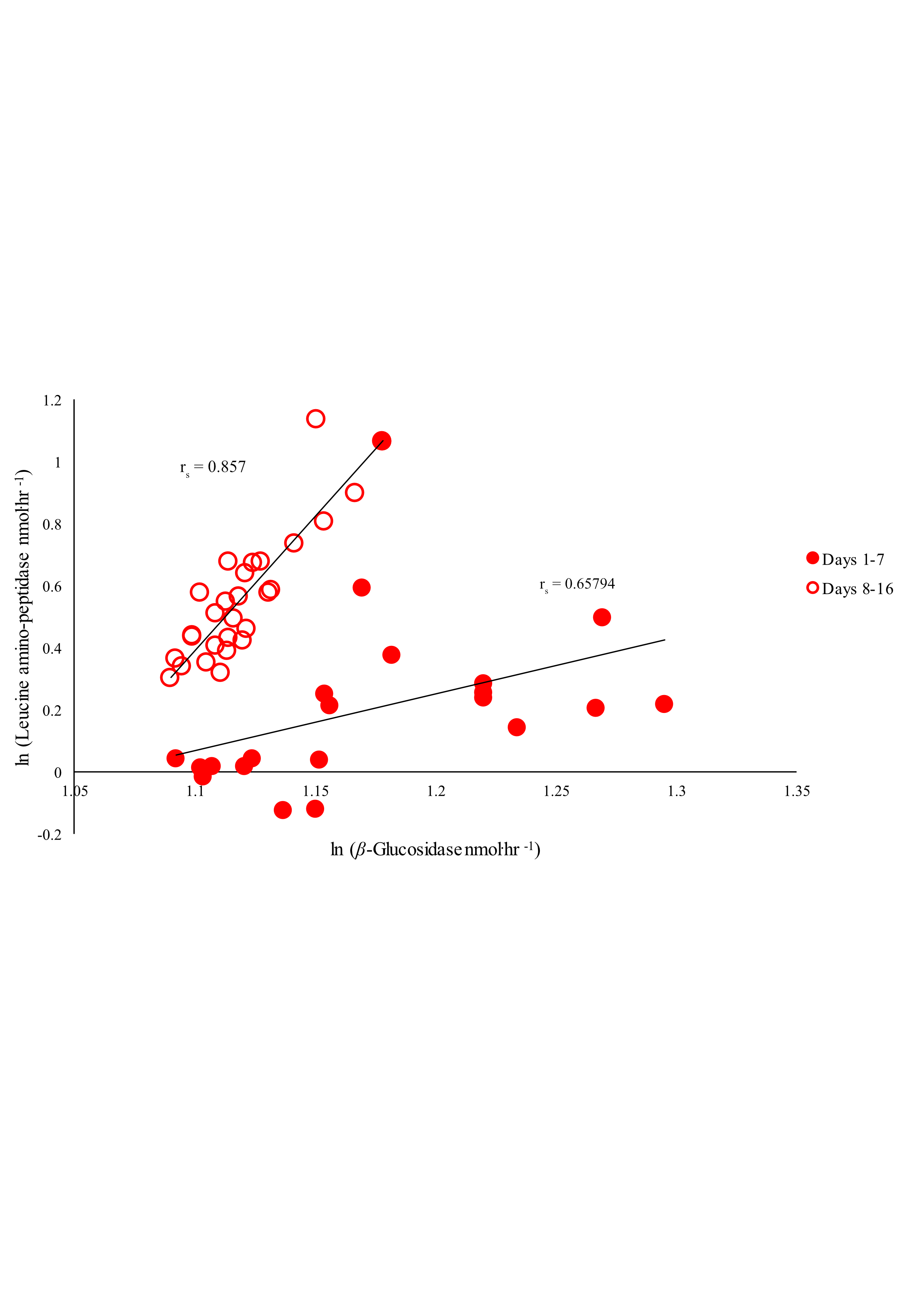

Supplement: FIG S2 [file mSystems.00290-20-sf002.tif]

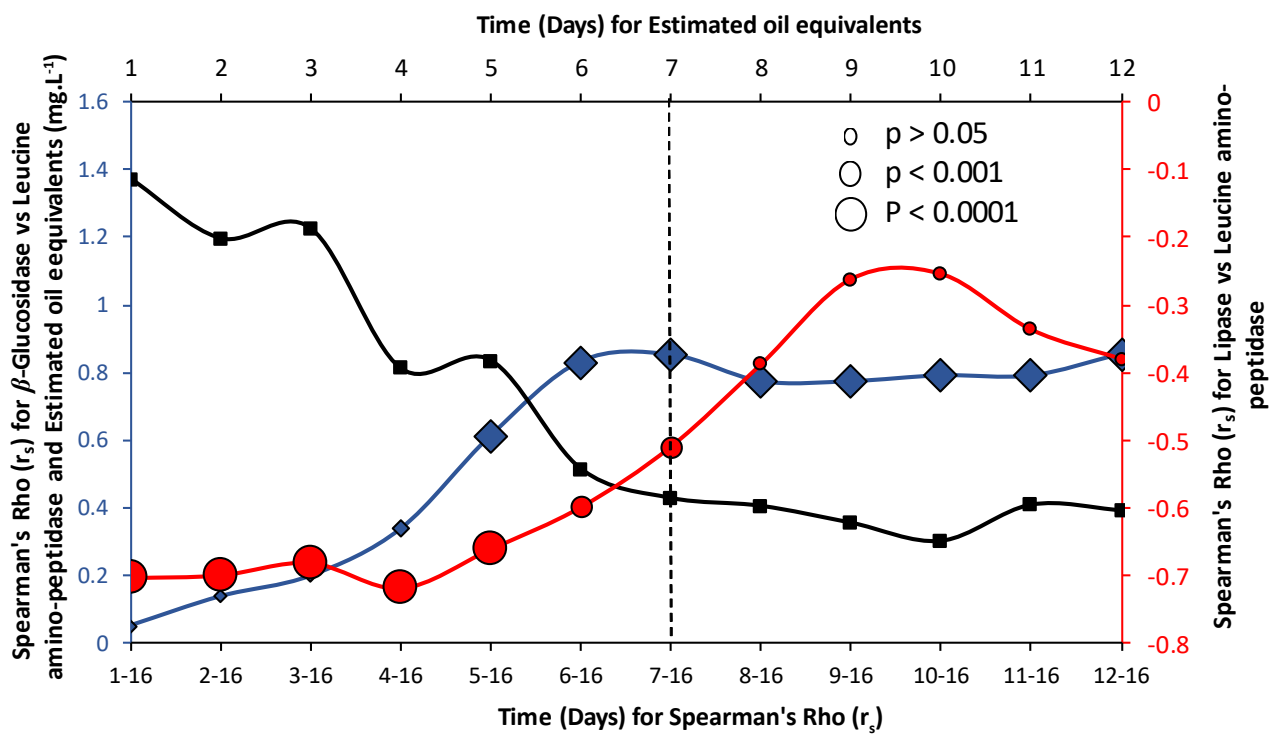

Supplement: FIG S3 [file mSystems.00290-20-sf003.pdf]

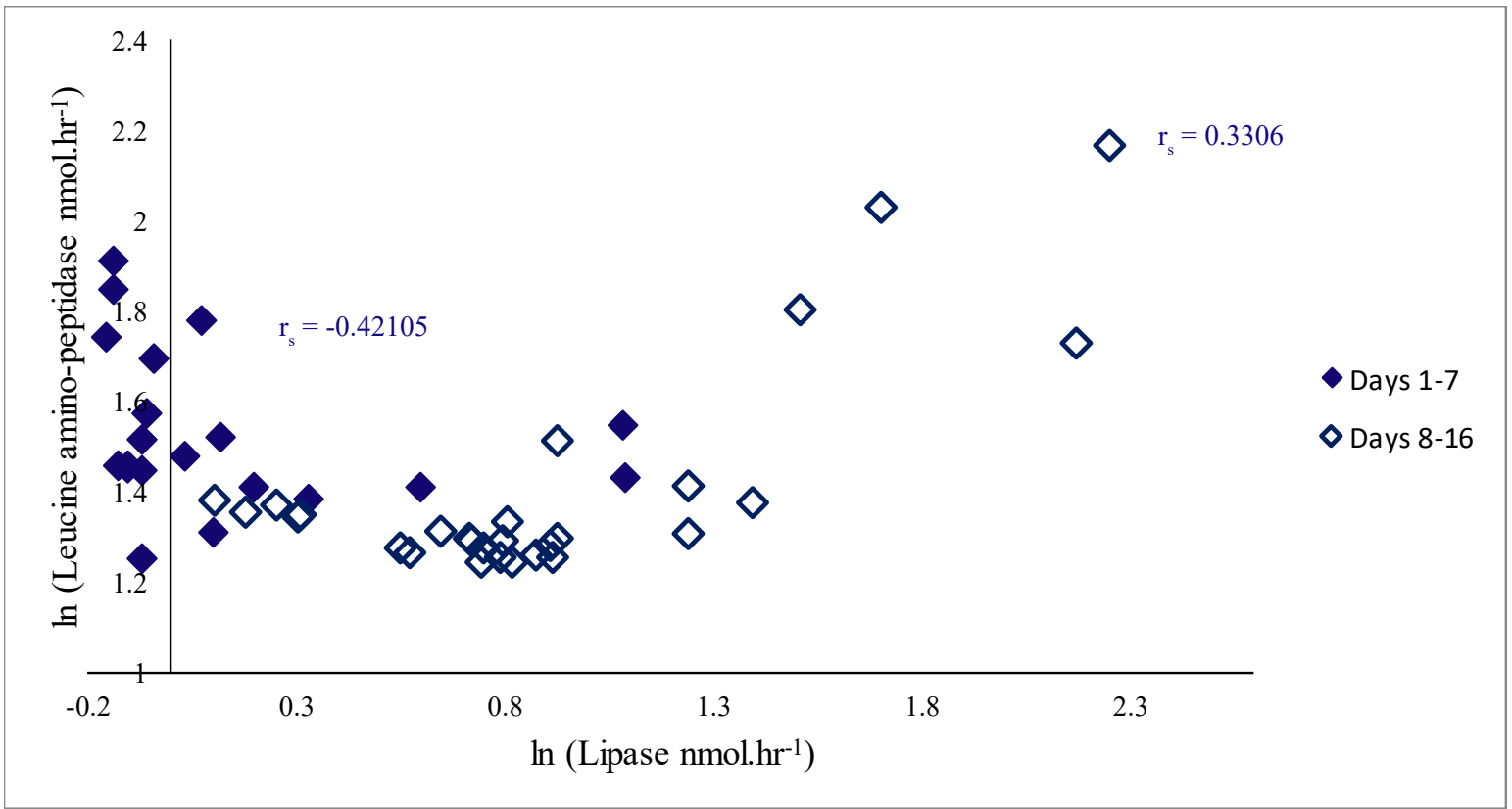

Supplement: FIG S4 [file mSystems.00290-20-sf004.pdf]

Relative Abundance (Order > 3.5%)

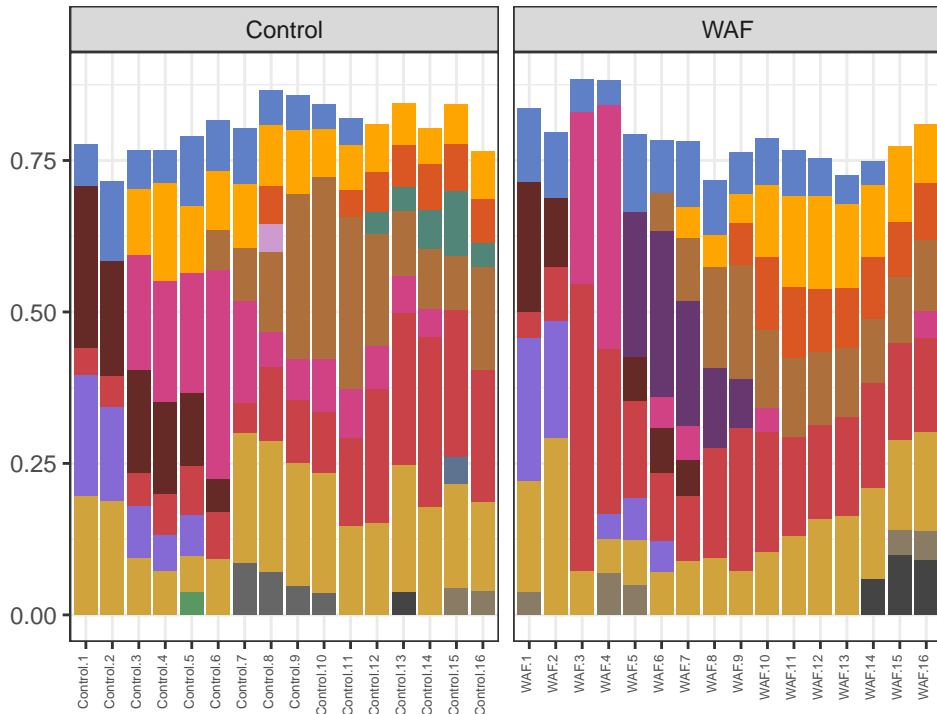

Supplement: FIG S5 [file mSystems.00290-20-sf005.pdf]
