# Supplementary material for: Exoenzymes as a Signature of Microbial Response to Marine Environmental Conditions
Source: mSystems. 2020 Apr 14;5(2):e00290-20. doi: 10.1128/mSystems.00290-20 (PMC7159900; doi:10.1128/mSystems.00290-20)
Supplement: TABLE S1 [file mSystems.00290-20-st001.docx]

|  | **POC** | **Glucosidase** | **Peptidase** | **Lipase** | **Phosphatase** | **Uronic acids** | **Neutral sugars** | **Proteins** | **COC** | **CON** |
| --- | --- | --- | --- | --- | --- | --- | --- | --- | --- | --- |
| **POC** | 1  (0.00) | 0.77  (0.00) | 0.3  (1.00) | 0.16  (1.00) | 0.06  (1.00) | 0.17  (1.00) | 0.16  (1.00) | 0.17  (1.00) | 0.06  (1.00) | -0.05  (1.00) |
| **Glucosidase** | 0.77  (0.00) | 1  (0.00) | 0.37  (0.56) | 0.22  (1.00) | 0  (1.00) | -0.05  (1.00) | -0.04  (1.00) | 0.03  (1.00) | -0.04  (1.00) | -0.09  (1.00) |
| **Peptidase** | 0.3  (0.04) | 0.37  (0.01) | 1  (0.00) | -0.66  (0.00) | 0.73  (0.00) | 0.48  (0.00) | 0.69  (0.00) | 0.72  (0.00) | 0.72  (0.00) | 0.67  (0.00) |
| **Lipase** | 0.16  (0.28) | 0.22  (0.13) | -0.66  (0.00) | 1  (0.00) | -0.71  (0.00) | -0.52  (0.00) | -0.79  (0.00) | -0.68  (0.00) | -0.79  (0.00) | -0.76  (0.00) |
| **Phosphatase** | 0.06  (0.71) | 0  (1.00) | 0.73  (0.00) | -0.71  (0.00) | 1  (0.00) | 0.52  (0.00) | 0.71  (0.00) | 0.77  (0.00) | 0.79  (0.00) | 0.71  (0.00) |
| **Uronic acids** | 0.17  (0.25) | -0.05  (0.72) | 0.48  (0.00) | -0.52  (0.00) | 0.52  (0.00) | 1  (0.00) | 0.72  (0.00) | 0.72  (0.00) | 0.67  (0.00) | 0.61  (0.00) |
| **Neutral sugars** | 0.16  (0.27) | -0.04  (0.79) | 0.69  (0.00) | -0.79  (0.00) | 0.71  (0.00) | 0.72  (0.00) | 1  (0.00) | 0.88  (0.00) | 0.91  (0.00) | 0.86  (0.00) |
| **Proteins** | 0.17  (0.26) | 0.03  (0.84) | 0.72  (0.00) | -0.68  (0.00) | 0.77  (0.00) | 0.72  (0.00) | 0.88  (0.00) | 1  (0.00) | 0.91  (0.00) | 0.87  (0.00) |
| **COC** | 0.06  (0.68) | -0.04  (0.78) | 0.72  (0.00) | -0.79  (0.00) | 0.79  (0.00) | 0.67  (0.00) | 0.91  (0.00) | 0.91  (0.00) | 1  (0.00) | 0.93  (0.00) |
| **CON** | -0.05  (0.76) | -0.09  (0.53) | 0.67  (0.00) | -0.76  (0.00) | 0.71  (0.00) | 0.61  (0.00) | 0.86  (0.00) | 0.87  (0.00) | 0.93  (0.00) | 1  (0.00) |

**Supplementary Table 1:** Summary statistics of correlation matrix analysis for POC, *β*-Glucosidase, leucine amino-peptidase (LAP), lipase, alkaline phosphatase (AP), total enzyme activities, uronic acids, neutral sugars, proteins, total EPS colloidal organic carbon (COC), and colloidal organic nitrogen (CON).
